# Biological network inferences for a protection mechanism against familial Creutzfeldt-Jakob disease with E200K pathogenic mutation

**DOI:** 10.1186/1755-8794-7-52

**Published:** 2014-08-22

**Authors:** Sol Moe Lee, Myungguen Chung, Kyu Jam Hwang, Young Ran Ju, Jae Wook Hyeon, Jun-Sun Park, Chi-Kyeong Kim, Sangho Choi, Jeongmin Lee, Su Yeon Kim

**Affiliations:** 1 Division of Zoonoses, Center for Immunology and Pathology, National Institute of Health, Korea Center for Disease Control and Prevention, Cheongju-si 363-700, Korea; 2 Division of Bio-Medical Informatics, Center for Genome Science, National Institute of Health, Korea Center for Disease Control and Prevention, Cheongju-si 363-700, Korea; 3 Department of Agricultural Biotechnology, Animal Biotechnology Major, Seoul National University, Seoul 151-921, Korea; 4 Division of Molecular and Life science, Hanyang University, Seoul 133-791, Korea

**Keywords:** PRNP, E200K-dependent fCJD, 85-year-old non-CJD individual with the E200K, Whole exome sequencing, Biological network analysis, Candidate protective factor against fCJD with E200K

## Abstract

**Background:**

Human prion diseases are caused by abnormal accumulation of misfolded prion protein in the brain tissue. Inherited prion diseases, including familial Creutzfeldt-Jakob disease (fCJD), are associated with mutations of the prion protein gene (*PRNP*). The glutamate (E)-to-lysine (K) substitution at codon 200 (E200K) in PRNP is the most common pathogenic mutation causing fCJD, but the E200K pathogenic mutation alone is regarded insufficient to cause prion diseases; thus, additional unidentified factors are proposed to explain the penetrance of E200K-dependent fCJD. Here, exome differences and biological network analysis between fCJD patients with E200K and healthy individuals, including a non-CJD individual with E200K, were analysed to gain new insights into possible mechanisms for CJD in individuals carrying E200K.

**Methods:**

Exome sequencing of the three CJD patients with E200K and 11 of the family of one patient (case1) were performed using the Illumina HiSeq 2000. The exome sequences of 24 Healthy Koreans were used as control. The bioinformatic analysis of the exome sequences was performed using the CLC Genomics Workbench v5.5. Sanger sequencing for variants validation was processed using a BigDye Terminator Cycle Sequencing Kit and an ABI 3730xl automated sequencer. Biological networks were created using Cytoscape (v2.8.3 and v3.0.2) and Pathway Studio 9.0 software.

**Results:**

Nineteen sites were only observed in healthy individuals. Four proteins (NRXN2, KLKB1, KARS, and LAMA3) that harbour rarely observed single-nucleotide variants showed biological interactions that are associated with prion diseases and/or prion protein in our biological network analysis.

**Conclusion:**

Through this study, we confirmed that individuals can have a CJD-free life, even if they carry a pathogenic E200K mutation. Our research provides a possible mechanism that involves a candidate protective factor; this could be exploited to prevent fCJD onset in individuals carrying E200K.

## Background

Transmissible spongiform encephalopathies (TSE), also called prion diseases, are rare fatal neurodegenerative disorders that affect humans and animals. TSE are characterized at the pathological level by abnormal accumulation of misfolding prion protein (PrP^sc^) affecting the central nervous system (CNS) [1,2]. In most cases, transmission of PrP^sc^ has been proven in animal models including primates and rodents; this has established PrP^sc^ as the transmissible agent of diseases [3-5]. In humans, the causative agent is encoded by the prion protein gene (*PRNP*) on chromosome 20p13. The incubation period for prion diseases observed in humans and animals is influenced by polymorphisms or mutations in *PRNP*. Pathogenic mutations in prion proteins that cause inherited prion diseases (IPD) are responsible for 10–15% of cases [6-8]. IPD have traditionally been classified as familial Creutzfeldt-Jakob disease (fCJD), fatal familial insomnia (FFI), and Gerstmann-Straussler-Scheinker syndrome (GSS). P102L, P105L, A117V, Y145stop, D178N, V180I, F198S, E200K, and V203I mutations in prion proteins are highly correlated with the pathogenicity of IPD [3-5,9-22].

The E200K substitution within PRNP is a major causative mutation of IPD [6,23]. The clinical features of fCJD patients with E200K are similar to those of sporadic CJD patients, and the median age of onset of CJD patients with E200K is 58, with a median disease duration of seven months [24]. The E200K mutation was first reported in 1989, and has since been found in North American Caucasians, Slovakians, Polish, German, Tunisian Jewish, Greek, Libyan Jews, and Chilean populations [13,25]. Although E200K is reportedly a causative mutation for fCJD, not all cases have a familial history. The mutation has also been observed in healthy individuals who are already above the median age for E200K-associated CJD; remarkably, in one case, an individual over 80 years of age with the E200K mutant died of non-CJD related causes [26,27].

The reported penetrance for CJD with E200K is 56–59.5% in individuals below 80 years of age, and, depending on the population under study, rises to 80–100% in individuals above 80 years of age [13,27-29]. This demonstrates that most of the individuals with E200K can be regarded as suffering from CJD after the median age, but that some individuals appear to survive without the symptoms of CJD. This has led scientists to propose that genetic background can contribute to the delayed onset of CJD [26].

A Korean CJD patient with E200K who was presented to us was presumed to represent a sporadic case, since there was no previous family history of CJD [30]. Three generations of the patient’s family (n = 12) provided us with their epidemiological details and whole blood samples; we investigated the PRNP genotypes of this pedigree and found that all of them were 129M homozygotes. E200K was observed in eight members of the family, including the patient. Five were 200E homozygotes, and 200K homozygotes were not observed.

Interestingly, the mother of the proband (born in 1927) was E200K heterozygous, but she did not have any symptoms related to neurodegenerative disorders, despite the fact that she was well over the median age for disease onset. This presentation makes the case extremely rare globally, and it is the first such case in Korea. We decided that follow-up studies of this family might further our understanding not only of the mechanisms by which E200K causes CJD, but also identify clusters of genetic polymorphisms that underlie neuroprotection.

Here, we report the genotype patterns and differences in the exome sequences between the above-mentioned family, CJD patients with E200K, and healthy individuals. We also discuss the biological network analysis undertaken to infer relationships between genotypes and prion disease onset.

## Results and discussion

Two types of variant filtering strategies were used (see Methods). Validation studies revealed that 19 of 24 sites were validated as SNVs, with the rest being false positive (Table 1). Primer sequences of 24 sites are listed in Additional file 1: Table S1. All 19 validated SNVs were detected on autosomes. The biological data and official full name associated with each protein of the 19 SNVs-containing genes are listed in Additional file 2: Table S2 and Additional file 3: Table S3. None of the genes have previously been associated with prion diseases, with the exception of NRXN2, which was reported to be down-regulated in mice infected with PrP^sc^[31]. Although the second variant filtering strategy was more relaxed than the first, only five variants met the criteria. However, all of them were confirmed as false positives. There are at least three hypothetical reasons to explain this. First, E200K may be the sole pathogenic mutation associated with CJD in these individuals. Second, CJD patients with E200K may indeed carry additional pathology-related mutations, but these may be already published, rather than de novo mutations. Third, the fact that we selected individuals with the E200K mutation in the first filtering strategy might influence the analysis with respect to prion disease onset or incubation period. Specifically, this may imply that the 85-year-old non-CJD individual with the E200K mutation might also carry protective SNVs at other loci. E200K is unlikely to be the sole mutation that determines CJD onset, since there are some elderly individuals (age, up to 85 years) in this study. CJD is a rare neurodegenerative disorder, and there is a small possibility of disease identification in elderly individuals who carry E200K mutation and are healthy. Thus, factors that either cause or protect against CJD might be discovered by comparing the exome of such individuals to other groups. In light of these considerations, we discarded the first two hypotheses and instead focused on the third possibility.

**Table 1:** The information of the 24 sites that were directly sequenced and the genotypes of analysed individuals

				**PRNP mutation**	**E200K**	**E200K**	**E200K**	**E200K**	**WT**	**E200K**	**E200K**	**E200K**	**WT**	**E200K**	**WT**	**E200K**	**WT**	**E200K**	**WT**	**WT**	**WT**	**WT**	**WT**	**WT**	**E219K**	**WT**	**WT**	**WT**	**WT**
				**Sample ID**	**Patient no. 1**	**Patient no. 2**	**Patient no. 3**	**Family no. 1**	**Family no. 2**	**Family no. 3**	**Family no. 4**	**Family no. 5**	**Family no. 6**	**Family no. 7**	**Family no. 8**	**Family no. 9**	**Family no. 10**	**Family no. 11**	**Family no. 12**	**Normal no. 1**	**Normal no. 2**	**Normal no. 3**	**Normal no. 4**	**Normal no. 5**	**Normal no. 6**	**Normal no. 7**	**Normal no. 8**	**Normal no. 9**	**Normal no. 10**
Variants filtering strategy	Chr.	Position	Gene	Year of birth Ref./Obs.	1948	non-descript	1935	1927	1950	1952	1955	1957	1962	1969	1971	1975	1977	1981	1983	1969	1975	1976	1977	1980	1982	1983	1983	1984	1987
Filtering strategy 1	1	27,268,000	NUDC	G/A	GG	GG	GG	G*A*	GG	G*A*	G*A*	G*A*	GG	GG	GG	GG	GG	G*A*	GG	GG	GG	GG	GG	GG	GG	GG	GG	GG	GG
	1	42,049,603	HIVEP3	C/T	CC	CC	CC	C*T*	C*T*	CC	CC	CC	C*T*	CC	CC	CC	CC	CC	CC	CC	C*T*	CC	CC	CC	CC	CC	CC	CC	CC
	2	64,199,317	VPS54	G/A	GG	GG	GG	G*A*	GG	GG	G*A*	G*A*	G*A*	GG	GG	GG	G*A*	G*A*	GG	GG	GG	G*A*	GG	GG	GG	GG	GG	GG	GG
	2	233,346,498	ECEL1	C/T	CC	CC	CC	C*T*	CC	CC	CC	CC	C*T*	CC	CC	CC	CC	CC	CC	CC	C*T*	CC	CC	C*T*	C*T*	CC	CC	CC	CC
	3	124,896,625	SLC12A8	A/G	AA	AA	AA	A*G*	A*G*	AA	A*G*	A*G*	AA	AA	AA	AA	A*G*	A*G*	A*G*	AA	AA	AA	AA	AA	AA	AA	AA	AA	AA
	4	187,153,290	KLKB1	C/T	CC	CC	CC	C*T*	CC	CC	C*T*	CC	CC	CC	CC	CC	CC	CC	CC	CC	CC	CC	CC	CC	CC	CC	CC	CC	CC
	5	139,884,478	ANKHD1-EIF4EBP3	G/C	GG	GG	GG	G*C*	GG	GG	GG	GG	G*C*	GG	GG	GG	GG	GG	GG	GG	GG	GG	GG	GG	GG	GG	GG	GG	GG
	6	159,185,617	SYTL3	T/C	TT	TT	TT	T*C*	T*C*	T*C*	TT	T*C*	TT	TT	TT	TT	TT	T*C*	T*C*	TT	TT	TT	TT	TT	TT	TT	TT	TT	TT
	8	2,088,717	MYOM2	G/T	GG	GG	GG	G*T*	G*T*	G*T*	G*T*	GG	G*T*	GG	GG	GG	G*T*	GG	GG	GG	GG	GG	GG	GG	GG	GG	GG	GG	GG
	9	18,950,859	FAM154A	C/T	CC	CC	CC	C*T*	C*T*	C*T*	C*T*	CC	C*T*	CC	CC	CC	CC	CC	CC	CC	CC	CC	CC	CC	CC	CC	CC	CC	CC
	10	24,831,649	KIAA1217	C/T	CC	CC	CC	C*T*	C*T*	C*T*	CC	CC	CC	CC	CC	CC	CC	CC	CC	CC	CC	CC	CC	CC	CC	CC	CC	CC	CC
	11	27,016,411	FIBIN	A/G	AA	AA	AA	A*G*	FL	AA	A*G*	A*G*	A*G*	AA	AA	AA	AA	A*G*	AA	AA	AA	A*G*	AA	AA	AA	AA	AA	AA	A*G*
	11	36,250,774	LDLRAD3	G/T	GG	GG	GG	G*T*	GG	G*T*	GG	GG	GG	GG	GG	GG	GG	GG	GG	GG	GG	GG	GG	GG	GG	GG	GG	GG	GG
	11	64,453,195	NRXN2	C/A	CC	CC	CC	C*A*	CC	C*A*	C*A*	C*A*	CC	CC	CC	CC	CC	C*A*	CC	CC	CC	CC	CC	CC	CC	CC	CC	CC	CC
	13	39,588,100	PROSER1	G/A	GG	GG	GG	G*A*	GG	GG	GG	GG	GG	GG	GG	GG	GG	GG	GG	GG	GG	GG	GG	GG	GG	GG	GG	GG	GG
	16	75,669,878	KARS	A/G	AA	AA	AA	A*G*	A*G*	AA	AA	A*G*	AA	AA	AA	AA	AA	AA	A*G*	AA	AA	AA	AA	AA	AA	AA	AA	AA	AA
	17	59,489,425	C17orf82	C/G	CC	CC	CC	C*G*	C*G*	C*G*	CC	C*G*	C*G*	CC	CC	CC	CC	CC	C*G*	CC	CC	CC	CC	CC	CC	CC	CC	CC	CC
	18	21,485,578	LAMA3	G/C	GG	GG	GG	G*C*	G*C*	G*C*	G*C*	GG	G*C*	GG	GG	GG	GG	GG	GG	GG	GG	GG	GG	GG	GG	GG	GG	GG	GG
	19	4,359,190	MPND	C/T	CC	CC	CC	C*T*	C*T*	C*T*	CC	CC	CC	CC	CC	CC	CC	CC	CC	CC	CC	CC	CC	CC	CC	CC	CC	CC	CC
Filtering strategy 2	17	45,219,336	CDC27	T/A	TT	TT	TT	TT	TT	TT	TT	TT	TT	TT	TT	TT	TT	TT	TT	TT	TT	TT	TT	TT	TT	TT	TT	TT	TT
	19	501,786	MADCAM1	C/A	CC	CC	CC	CC	CC	CC	CC	CC	CC	CC	CC	CC	CC	CC	CC	CC	CC	CC	CC	CC	CC	CC	CC	CC	CC
	19	50,510,999	VRK3	A/T	TT	TT	TT	TT	TT	TT	TT	TT	TT	TT	TT	TT	TT	TT	TT	TT	TT	TT	TT	TT	TT	TT	TT	TT	TT
	19	50,511,000		C/T	TT	TT	TT	TT	TT	TT	TT	TT	TT	TT	TT	TT	TT	TT	TT	TT	TT	TT	TT	TT	TT	TT	TT	TT	TT
	19	52,096,053	AC018755.11	T/A	T*A*	T*A*	T*A*	AA	T*A*	T*A*	T*A*	T*A*	T*A*	AA	T*A*	TT	T*A*	T*A*	TA	AA	T*A*	T*A*	TT	AA	TT	T*A*	T*A*	T*A*	TT

The 63-year-old proband (Case 1) is the eldest progeny of an 84-year-old non-CJD individual with the E200K mutation (Family Member 1, hereafter FM1); the rest of the progeny are younger than the proband by about 2–17 years. The 19 validated SNVs were not found in the proband and their sibling, which is expected as all mutations observed in the proband were filtered during the first filtering strategy. Between 5 and 10 variations were observed in the other siblings. FM7 and FM9 are heterozygous for E200K, and FM8 is homozygous for the wild-type E200 variant. Nucleotide variants in all three CJD patients with E200K were homozygous, and identical to the reference nucleotide sequence in dbSNP 132; the single-nucleotide variations (SNVs) in the mother of the proband were heterozygous and contained SNVs that have not been previously reported for dbSNP 132. Nucleotide variations in *PROSER1* were only observed in FM1, but the biological functions of this proline and serine-rich protein are unknown. We also found SNVs in *NUDC*, *KLKB1*, and *NRXN2*; these were specifically present in non-CJD E200K individuals, but not in CJD patients with E200K counterparts. SNVs in 10 healthy individuals with no family history of CJD were observed in four genes (*HIVEP3*, *VPS54*, *ECEL1*, and *FIBIN*). Thus, these four SNVs were considered as relatively common among the general Korean population.

**Figure 1:**
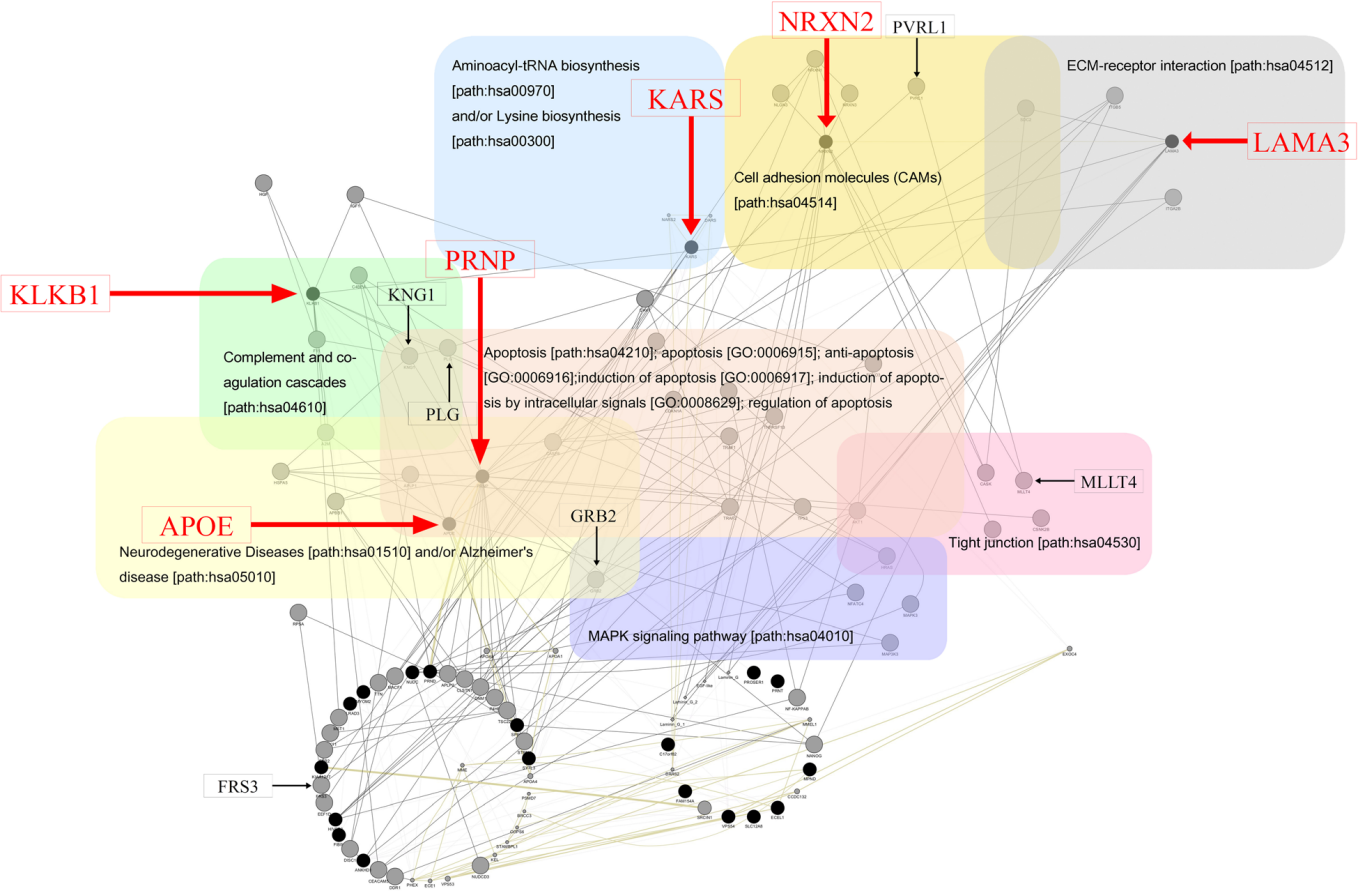
**Interaction network among 24 seed nodes (marked as black circle) and their 80 interacted nodes (marked as grey circle) using three Cytoscape plug-ins.** Six seed nodes (KLKB1, KARS, NRXN2, LAMA3, PRNP, and APOE) clustered according to the KEGG pathway criteria were annotated by red squares and indicated by red arrows. The six interaction nodes with seed node were annotated by black squares and indicated by black arrows. The apoptosis cluster (path: hsa04210) was expanded using GO data [apoptosis (GO: 0006915), anti-apoptosis (GO: 0006916), induction of apoptosis (GO: 0006917), induction of apoptosis by intracellular signals (GO: 0008629), regulation of apoptosis (GO: 0042981), and positive regulation of apoptosis (GO: 0043065)].

We used biological networking programs to gain new insight into possible mechanisms that protect individuals carrying E200K against CJD. The 19 proteins associated with SNVs in our study, and 5 proteins that are reported as prion disease-related, such as PRNP, prion protein doublet (PRND), prion protein testis-specific (PRNT), shadow of prion protein (SPRN), and apolipoprotein E (APOE), were used as seed proteins.A total of 104 nodes, including 24 seed nodes, were identified (Figure 1). The average number of neighbours of nodes was 4.327, with the exception of PRNT, which was isolated from the others. Among the 19 proteins harbouring validated SNVs, only four of them (KLKB1, KARS, NRXN2, and LAMA3) were clustered by Kyoto Encyclopedia of Genes and Genomes (KEGG) pathway criteria.

PRNP and APOE, which are within the neurodegenerative disorder and apoptosis-related seed nodes, were linked with Kininogen1 (KNG1) and plasminogen (PLG), which are apoptosis- and complement and coagulation cascade-related proteins [32-34]. KNG1 was linked with KLKB1, which was one of the seed nodes [35-39]. LAMA3 was also linked with PLG in our study. Consistent with this, LAMA3 is reported to contain a cleavage site for plasmin, the activated form of PLG [40].

PRNP has been previously linked with growth factor receptor bound protein 2 (GRB2) [41]. GRB2 is related to proteins that are implicated in neurodegenerative diseases (path: hsa01510), MAPK-signalling pathway (path: hsa04010), and Huntington's disease (path: hsa05040). GRB2 directly interacts with FRS3 [42], which is itself related to proteins involved in the fibroblast growth factor receptor signalling pathway, and signal transduction (GO: 0008543 and 0007165, respectively). FRS3 interacts with KARS, which is one of the seed proteins in the current study [43]. However, only a low-level biological interaction between FRS3 and KARS was reported. Hence, the relevance of KARS to prion disease at this stage remains unclear.

**Figure 2:**
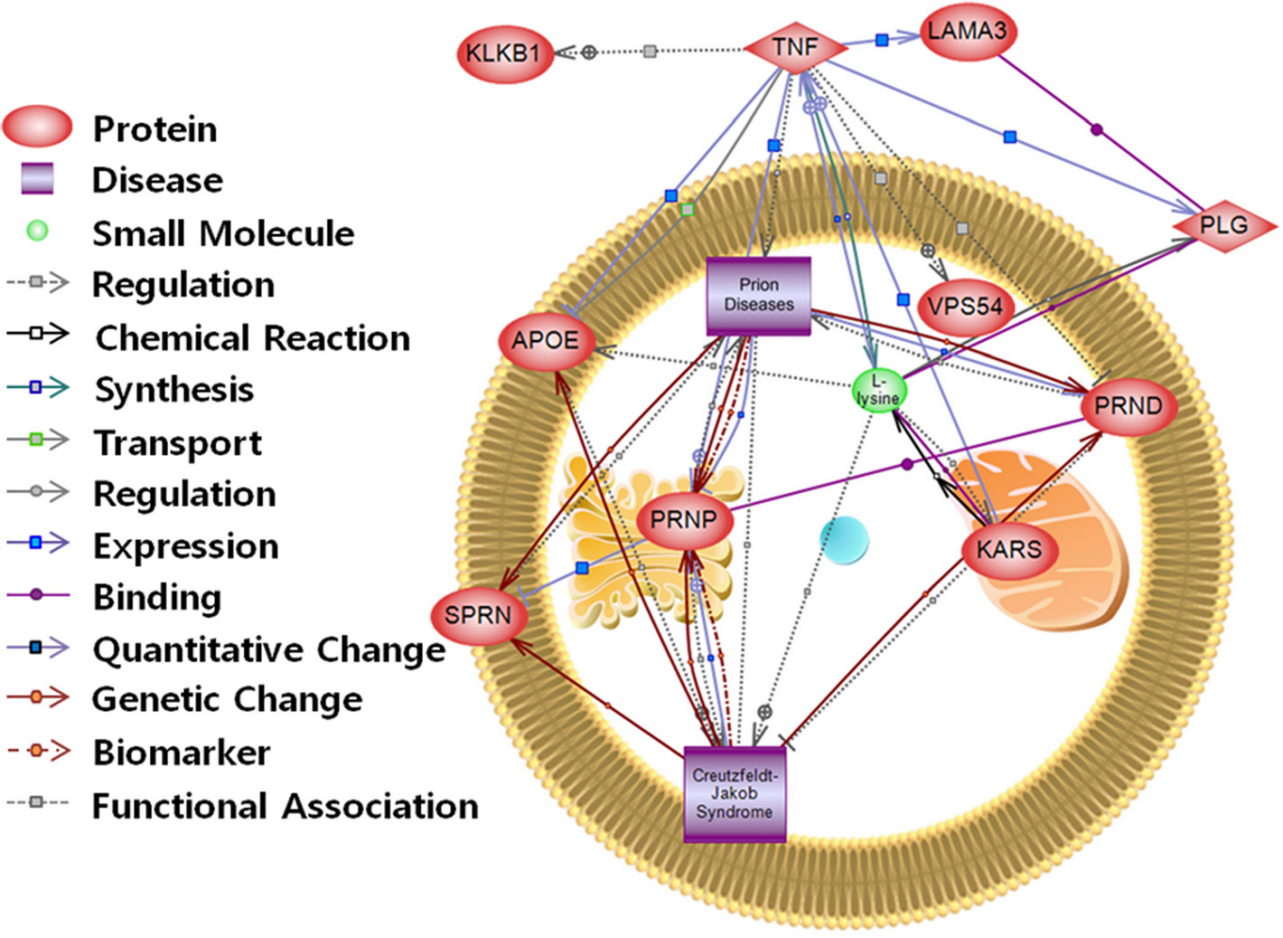
**Interaction network using Pathway studio.** Interactions between nodes are denoted by coloured arrows or lines. Seed nodes are marked as red circles. PLG and TNF are shown as significant interactions with the seed nodes, PRNP, prion diseases were marked as diamond-square.

Interaction between PRNP and PVRL1 (also called PRR) was observed, although a strong relationship between the two was not evident from the publication [44]. Although PRNP was previously associated with cell adhesion molecules (CAMs) such as neural CAM1 (N-CAM1), neural CAM2 (N-CAM2) and neural adhesion molecule F3 (contactin1) [44], there are no reports of a direct relationship between PRNP and PVRL1. Thus, we believe the PRNP/PVRL1 interaction was false. PVRL1 was linked with MLLT4, and a previous report says that it interacts with MLLT4 (also called Afadin), a PDZ domain-containing protein [45-47]. MLLT4 was linked to NRXN2, which is one of the seed proteins in this study [48]. It is known that C-terminal peptides of the NRXN2 sequence can interact with the PDZ domain of MLLT4.Biological network analysis using Pathway studio v9 identified four proteins, namely, KLKB1, LAMA3, KARS, and VPS54 (Figure 2). VPS54 and KLKB1 were identified since they have interactions with tumour necrosis factor (TNF), rather than with prion diseases and/or prion protein; they were thus excluded from the network analysis. LAMA3 and KARS were identified because of indirect interaction with prion protein and prion diseases; they were therefore subjected to follow-up analysis to determine whether they were associated with mechanisms that protect against prion disease.

*LAMA3* is located at 18q11.2, and a de novo mutation (K2180N) was observed in this study. The C-terminal part of LAMA3 forms a compact globular domain, called the G domain, which is divided into subdomains G1–G5; the G1 subdomain is a plasmin-binding site, and the G4 subdomain contains a plasmin cleavage site [40]. PLG is reported to bind a lysine-rich portion of PrP, and in doing so can stimulate propagation of PrP^sc^. l-Lysine has a role in blocking plasminogen-mediated stimulation of PrP^sc^ propagation [49,50].

Aminoacyl-tRNA synthetases (ARSs) ligate specific amino acids to their cognate tRNAs for protein synthesis, and human lysyl-tRNA synthetase (KARS) are the one of the ARSs for lysine. The Y201H mutation in KARS was detected as a de novo mutant; however, this mutation has previously been published in dbSNP build 134 as rs150529876. However, rs150529876 is scarcely found in East Asian populations referred to in the 1000 Genome allele frequencies data, which confirms that it is a very rarely detected variant in human population. The Y201H mutation might alter the levels of lysine in the human body, and may favour the interaction between PLG and lysine, instead of with PrP^sc^. Possibly, this might ultimately change the incubation period prior to CJD onset.

KARS is also known to induce secretion of the proinflammatory cytokine TNF from macrophages [51]. Lack of follicular dendritic cells (FDCs) was observed in TNF-deficient (TNF^-/-^) mice [52], and these animals do not accumulate scrapie in their spleens upon challenge with the ME7 scrapie strain [53]. This is likely since mature FDCs are required for PrP^sc^ aggregation. Thus, we suggest that the Y201H mutation in KARS engenders a failure of TNF induction, which in turn prevents PrP^sc^ accumulation. We consider that this mutant form of KARS leads to an increased incubation period for prion diseases.

## Conclusions

In summary, we identified 19 SNVs that were differentially present in a healthy, 86-year-old individual with E200K, and in CJD patients with E200K. Each of these 19 SNVs is a candidate protective factor against E200K-associated CJD, since to date they were only observed in healthy individuals. However, longer follow-up studies of the family are required to definitively conclude that these 19 SNVs are CJD-protective factors. Our biological network analysis may also explain why FM1, who is the mother of the proband, does not have CJD symptoms, even though she has the E200K pathogenic mutation. Four proteins (NRXN2, KLKB1, KARS, and LAMA3) that harbour rarely observed SNVs have biological interactions that are associated with prion diseases and/or prion protein. However, we could not find significantly strong evidence that the other 15 proteins that harbour de novo SNVs had a biologically relevant association with prion diseases.

Through this study, we confirmed that individuals can have a CJD-free life, even if they harbour the pathogenic E200K mutation, and we provide possible protective mechanisms to explain this observation. Our research provides fundamental insight into the mechanisms that underlie the onset of prion disease, and suggests therapeutic strategies to treat E200K-associated CJD. We expect that ambiguous personal susceptibility on PRNP mutation causing fCJD can be defined through further studies of combined interpretation of biological network analysis derived from next-generation sequencing of individual genomes and clinical information.

## Methods

### Subjects

This study was approved by Institutional Review Board, Korea Centers for Disease Control and Prevention (IRB No. 2010-02CON-06-P). Written informed consent was obtained from the participants or legal guardian of each patient.

### Three CJD patients

All three CJD patients with E200K were described in this study, and they were South Korean natives. Case 1 was born in 1948, and was presumed to have developed symptoms in August 2011. The birth date of CJD Case 2 was not recorded. The patient first showed symptoms of CJD on January 8, 2011. Case 3 was born in 1935, and the patient first showed symptoms of CJD on March 14, 2010. All patients informed us that did not have a family history of CJD; thus, all three CJD patients with the E200K mutation were regarded as sporadic-like cases.

### 12 individuals in the family of case 1

**Figure 3:**
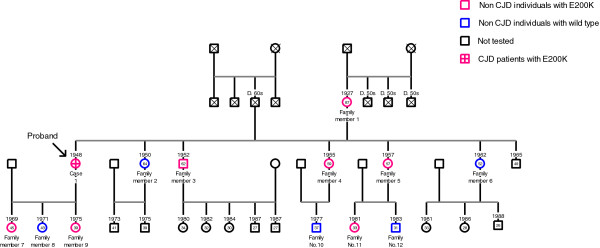
**Pedigree of a three-generation family of case 1.** Circles denote female subjects, squares denote male subjects, and symbols with diagonals denote dead subjects. The CJD allele status is explained in legend located in the upper right panel. The proband is marked with a black arrow. The age of each individual is noted in each symbol, and year of birth is noted upper part of each symbol. Died individuals were denoted as “X in a square” and their ages at death are shown above the square.

The three-generation family (n = 12) of case 1 generously contributed their time and materials to this study. The pedigree of the family was described using the GenoPro (http://www.genopro.com) pedigree drawing program (Figure 3).

### Healthy Korean exome sequences

The exome sequences of 14 healthy Koreans stored in Division of Center for Genome Science in KNIH were used as control for variation filtering.

### 10 randomly selected healthy individuals

Since DNA samples for 14 healthy Korean described above were not stored, DNA samples from 10 healthy individual volunteers in their 20s–40s were selected for variant validation.

### DNA extraction

gDNA samples were extracted from the individuals described above (except for 14 healthy Korean samples) for variant filtering. Total DNA was isolated from whole blood samples using QIAamp DNA Blood Mini Kit (QIAGEN, Korea), according to the manufacturer’s instructions. The extracted DNA was quantified using the Quant-iT PicoGreen dsDNA kit (Invitrogen, Carlsbad, CA).

### Exome sequencing

Exome sequencing of the three CJD patients with E200K and 11 of the three-generation family of Case 1 were performed using the Solexa sequencing technology platform (HiSeq2000, Illumina, San Diego, CA), following the manufacturer’s instructions. Exome sequencing of FM 7 was excluded since the quantity of gDNA (<1 μg) was not sufficient in this case.

### Exome capture libraries and sequencing

Three micrograms of the extracted DNA was randomly sheared using the Covaris System to generate ~150-bp inserts. The sonicated DNA was end-repaired using T4 DNA polymerase and Klenow polymerase, and Illumina paired-end adaptor oligonucleotides were ligated to the sticky ends. Ligated DNA was size-selected for lengths between 200 and 250 bp. The purified DNA library was hybridized with SureSelect Human All Exons probe set (Agilent, Santa Clara, CA) to capture 50-Mb targeted exons following the manufacturer’s instructions. Captured libraries were loaded onto the Illumina flow cell for sequencing on the Illumina HiSeq2000 instrument.

### Mapping and variations detection

Sequence reads mapping was performed using CLC Genomics Workbench v5.5 (CLC Bio, Aarhus, Denmark) with the human reference genome (GRCh37/hg19, dbSNP build 132). Variant calling was performed with default parameters. In the mapping steps, an average of 5.6 gigabases of uniquely mapping reads were obtained, and 3.6 gigabases of uniquely mapping reads were aligned on target per sample, with an average of 64% of all reads mapping on target. Approximately 86% of bases have basecall quality scores were over Q30 (Phred score of 30) per sample.

### Variants filtering

**Figure 4:**
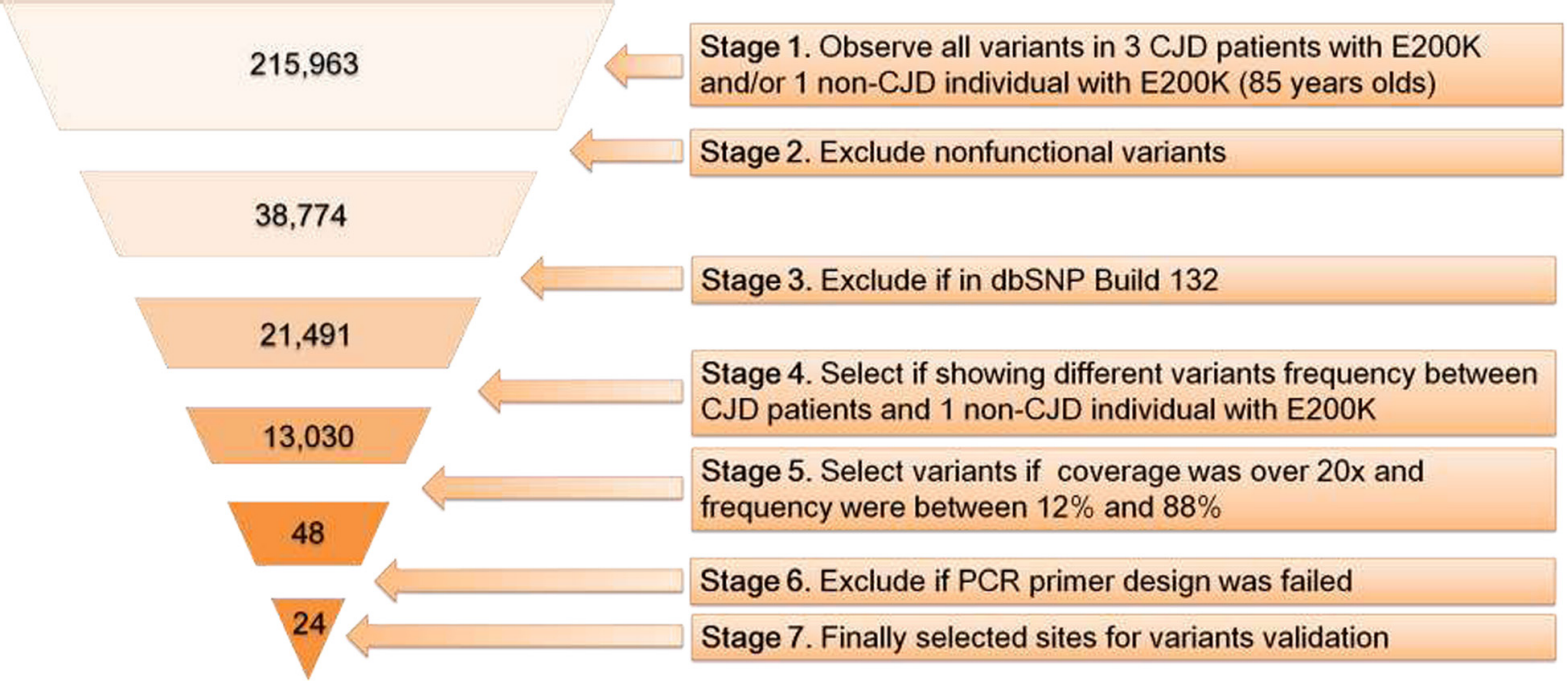
**Variant filtering strategy.** Overview of variant filtering of whole exome sequencing data.

Only variants showing different variation frequencies between CJD patients and non-CJD individuals were selected. Next, only non-synonymous variants were selected, and then variant filtering was performed based on the hypothesis that the mutation underlying familial CJD with E200K was rarely present in the general population (Figure 4). Nucleotide variants presented in dbSNP build 132 were filtered out. We then excluded variants having depth of coverage below 20×. Two types of variant filtering strategy were used. For the first filtering strategy, observed nucleotide variants in the 85-year-old non-CJD individual with E200K, but not observed in the three CJD patients with E200K. For the second filtering strategy, we only observed nucleotide variants in the three CJD patients with E200K. However, none of the sites were selected for the second strategy. Thus, the second filtering condition was more relaxed than that of the first. This meant that five variants were selected even though coverage was lower than 20×. A total of 48 sites were selected, but only 24 sites were analysed in the follow-up validation process, as we experienced PCR primer failures with the remaining samples.

### Validation of selected variants

PCR primer sets and their annealing temperature are described in Additional file 1: Table S1. The final volume of the PCR was 10 μl, consisting of 10 ng DNA, 0.5 μM each primer pair, 0.25 mM dNTPs, 3 mM MgCl_2_, 1 μl of 10× reaction buffer, and 0.25 U Taq DNA polymerase (Intron Biotechnology, Seongnam-Si, Gyeonggi-do, Korea). The PCR reaction was performed using 96-Well GeneAmp PCR System 9700 (Applied Biosystems, Foster City, CA) under the following conditions: initial denaturation at 94°C for 5 min, followed by 35 cycles of denaturation at 94°C for 30 s, annealing at 60°C for 30 s, initial extension at 72°C for 30–60 s, and final extension at 72°C for 10 min. The PCR products were purified using a MultiScreen 384-PCR Filter Plate (Millipore, Billerica, MA). The purified products were then sequenced using a BigDye Terminator Cycle Sequencing Kit and an ABI 3730xl automated sequencer (Applied Biosystems, Foster City, CA). The sequencing primers were the same as those used for the PCR amplification.

### Biological network analysis

1) Cytoscape (http://www.cytoscape.org/)

Cytoscape and three plug-ins, MiMI v3.1.1 (http://mimi.ncibi.org/MimiWeb/main-page.jsp), GeneMania v3.2.1 (http://www.genemania.org/), and Agilent Literature Search 3.0.3 beta (http://www.agilent.com/labs/research/litsearch.html) were used to construct a biological network. Due to compatibility issues, we used MiMI in Cytoscape v2.8.3 rather than v3.0.2. Next, all three networks were merged and visualized in Cytoscape v3.0.2. All nodes were clustered automatically using listed KEGG pathway criteria and the MiMI plug-in.

2) Pathway Studio v9 (Ariadne, Rockville, MD)

The second set of biological networks in this study was created using Pathway Studio 9.0 software (http://www.elsevier.com/online-tools/pathway-studio). The molecular interaction data were extracted by Elsevier's MedScan text-mining software, which contains almost 30 million biological articles and abstracts.

## Abbreviations

fCJD: Familial Creutzfeldt-Jakob disease; IPD: Inherited prion diseases; *PRNP*: Prion protein gene; SNVs: Single nucleotide variants; FFI: Fatal familial insomnia; GSS: Gertsmann-striussler-scheinker syndrome; FM: Family Member; PRND: Prion protein doublet; PRNT: Prion protein testis specific; SPRN: Shadow of prion protein; PCR: Polymerase chain reaction.

## Competing interests

The authors' declare that they have no competing interests.

## Authors’ contributions

SML, MC and SYK designed the study. SML and MC collected the data and performed data analysis. KJH, YRJ discussed and interpreted results. JWH and SYK collected epidemiological details and whole blood samples for the study. JSP, CKK, SC, and JL assisted with the analysis. SML, MC, and SYK wrote the manuscript. All authors read and approved the final manuscript.

## Pre-publication history

The pre-publication history for this paper can be accessed here:

http://www.biomedcentral.com/1755-8794/7/52/prepub

## Supplementary Material

Additional file 1: Table S1 Primer sequences for variants validation. Click here for file

Additional file 2: Table S2 Biological information of the 19 proteins harbouring the 19 validated sites. Known biological functions of the proteins were obtained from Kyoto Encyclopedia of Genes and Genomes (KEGG) and The Gene Ontology (GO) project (http://www.geneontology.org/). Click here for file

Additional file 3: Table S3 The information of 19 validated sites. Five sites that were false-positive were not recorded in this table. "-" means that there is no data for dbSNP 138. East Asians, Americans, Europeans, Africans were denoted as ASN, AMR, EUR, and AFR respectively. Chromosome, reference alleles and observed alleles in this study were denoted as Chr., Ref., and Obs. respectively. Click here for file
