# Supplementary material for: Biological network inferences for a protection mechanism against familial Creutzfeldt-Jakob disease with E200K pathogenic mutation
Source: BMC Med Genomics. 2014 Aug 22;7:52. doi: 10.1186/1755-8794-7-52 (PMC4151374; doi:10.1186/1755-8794-7-52)
Supplement: Additional file 1: Table S1 — Primer sequences for variants validation. [file 1755-8794-7-52-S1.doc]

Additional file 1: Table S1. Primer sequences for variants validation

| **No.** | **Fragment ID** | **Position (chr:physical position)** | **Gene** | **Primer sequence** | | **Tm** |
| --- | --- | --- | --- | --- | --- | --- |
| **1** | KNIHval-001 | 1:27,268,000 | NUDC | Forward Primer | acaggtgctttgcaggtat | 60℃ |
|  |  |  |  | Reverse Primer | CTCTAGCTGCAGCCTCTCT |  |
| **2** | KNIHval-002 | 1:42,049,603 | HIVEP3 | Forward Primer | AGATGAGGGTTCCACAAAT | 60℃ |
|  |  |  |  | Reverse Primer | GTGGCTTCTCCTTCAAGAC |  |
| **3** | KNIHval-003 | 2:64,199,317 | VPS54 | Forward Primer | ACATACCAACCACGAAGAT | 60℃ |
|  |  |  |  | Reverse Primer | CTGTTGCAAAACACTTTCA |  |
| **4** | KNIHval-004 | 2:233,346,498 | ECEL1 | Forward Primer | CTGAGACCCACCCTCACC | 60℃ |
|  |  |  |  | Reverse Primer | AGGTTCCACTGGCATTCAC |  |
| **5** | KNIHval-005 | 3:124,896,625 | SLC12A8 | Forward Primer | GGCAAACTCACACCTACAA | 60℃ |
|  |  |  |  | Reverse Primer | AATCGCATGTCAGTGTGTT |  |
| **6** | KNIHval-006 | 4:187,153,290 | KLKB1 | Forward Primer | CATTGTTCAAATAGTTGCCTTA | 60℃ |
|  |  |  |  | Reverse Primer | TGAAAAACAGCTTCTCCAA |  |
| **7** | KNIHval-007 | 5:139,884,478 | ANKHD1-EIF4EBP3 | Forward Primer | aggtctgtctgattccaaag | 60℃ |
|  |  |  |  | Reverse Primer | TTCCTTTCCAAAGAAGATCA |  |
| **8** | KNIHval-008 | 6:159,185,617 | SYTL3 | Forward Primer | GTGGTTGGCCTTTTAATGT | 60℃ |
|  |  |  |  | Reverse Primer | GATGGAGACTGCTCAAGGT |  |
| **9** | KNIHval-009 | 8:2,088,717 | MYOM2 | Forward Primer | GCAGCTTAGAGAAGGCTGT | 60℃ |
|  |  |  |  | Reverse Primer | CATATTGTATTCCCTCCCTTC |  |
| **10** | KNIHval-010 | 9:18,950,859 | FAM154A | Forward Primer | AATGCATTAGCACTGCACA | 60℃ |
|  |  |  |  | Reverse Primer | TGGCGGAAGTACTCTTAGG |  |
| **11** | KNIHval-011 | 10:24,831,649 | KIAA1217 | Forward Primer | CAGTTGGAGAACAAAGCAA | 60℃ |
|  |  |  |  | Reverse Primer | CCTGTGGTAACCTGAGAGG |  |
| **12** | KNIHval-012 | 11:27,016,411 | FIBIN | Forward Primer | GGGACTATGAGGAGAACGA | 60℃ |
|  |  |  |  | Reverse Primer | CCAGAAAGTCCTCGTTGA |  |
| **13** | KNIHval-013 | 11:36,250,774 | LDLRAD3 | Forward Primer | CTCAGAAATGGGCTGAAGT | 60℃ |
|  |  |  |  | Reverse Primer | AACCCATATGGACTTTGGA |  |
| **14** | KNIHval-014 | 11:64,453,195 | NRXN2 | Forward Primer | CCAACTCCTGTTCTTCTCC | 60℃ |
|  |  |  |  | Reverse Primer | TACGACCTGTCACACAACC |  |
| **15** | KNIHval-015 | 13:39,588,100 | PROSER1 | Forward Primer | AGCCAGCCCACTTGTGAC | 60℃ |
|  |  |  |  | Reverse Primer | TCACTGCCAGGTCCTTCTG |  |
| **16** | KNIHval-016 | 16:75,669,878 | KARS | Forward Primer | CCAAAAGCAGCAGTTAGAA | 60℃ |
|  |  |  |  | Reverse Primer | AGTTGAGTGAGGGTTCCAG |  |
| **17** | KNIHval-017 | 17:59,489,425 | C17orf82 | Forward Primer | CAGCTGACCGCGAACTCCT | 60℃ |
|  |  |  |  | Reverse Primer | GGAGGGTGCCCAGGAGAC |  |
| **18** | KNIHval-018 | 18:21,485,578 | LAMA3 | Forward Primer | CCCTTTGAACTTCATCCTT | 60℃ |
|  |  |  |  | Reverse Primer | TCATCTGCATTGACACTGA |  |
| **19** | KNIHval-019 | 19:4,359,190 | MPND | Forward Primer | CCTCTGGCTAAGGTCACTC | 60℃ |
|  |  |  |  | Reverse Primer | GACCAGGGTCACCAGTCT |  |
| **20** | KNIHval-020 | 17:45,219,336 | CDC27 | Forward Primer | GATCTTTTAGATCTAGCCTTCT | 60℃ |
|  |  |  |  | Reverse Primer | TTACAGTACCTTCATTGTTTTT |  |
| **21** | KNIHval-021 | 19:501,786 | MADCAM1 | Forward Primer | CTCACCAGAGTGGTCCAG | 60℃ |
|  |  |  |  | Reverse Primer | TTCATCCAAGGAACCTCTC |  |
| **22** | KNIHval-022 | 19:50,510,999, 50,511,000 | VRK3 | Forward Primer | ACTGTCCTGGTGCTGAGA | 60℃ |
|  |  |  |  | Reverse Primer | catagaaggtcccaggaaa |  |
| **23** | KNIHval-023 | 19:52,096,053 | AC018755.11 | Forward Primer | CAAAGGTGGACTCCCTATT | 60℃ |
|  |  |  |  | Reverse Primer | TCAACTGTCTGGGCTACAG |  |
