# Supplementary material for: Biological network inferences for a protection mechanism against familial Creutzfeldt-Jakob disease with E200K pathogenic mutation
Source: BMC Med Genomics. 2014 Aug 22;7:52. doi: 10.1186/1755-8794-7-52 (PMC4151374; doi:10.1186/1755-8794-7-52)
Supplement: Additional file 2: Table S2 — Biological information of the 19 proteins harbouring the 19 validated sites. Known biological functions of the proteins were obtained from Kyoto Encyclopedia of Genes and Genomes (KEGG) and The Gene Ontology (GO) project (http://www.geneontology.org/). [file 1755-8794-7-52-S2.doc]

- Additional file 2: Table S2. Biological information of the 19 proteins harboring the 19 validated sites. Known biological functions of the proteins were obtained from Kyoto Encyclopedia of Genes and Genomes (KEGG) and The Gene Ontology (GO) project (http://www.geneontology.org/).

| **No.** | **Official Symbol** | **Official full name** | **Also Known as** | **Location** | **Known biological function** | |
| --- | --- | --- | --- | --- | --- | --- |
|  |  |  |  |  | KEGG | GO |
| **1** | NUDC | nuclear distribution protein | HNUDC; MNUDC; NPD011 | 1p35-p34 | - | Protein binding, Cell cycle  Mitosis |
| **2** | HIVEP3 | human immunodeficiency virus type I enhancer binding protein 3 | KRC; KBP1; SHN3; ZAS3; KBP-1; ZNF40C; Schnurri-3 | 1p34 | - | DNA binding, Zinc ion binding, Metal ion binding |
| **3** | VPS54 | vacuolar protein sorting 54 homolog | WR; HCC8; SLP-8p; VPS54L; hVps54L | 2p13-p14 | - | protein transport  retrograde transport |
| **4** | ECEL1 | endothelin converting enzyme-like 1 | XCE; DA5D; DINE; ECEX | 2q37.1 | - | Metalloendopeptidase activity, Proteolysis, Neuropeptide signaling pathway |
| **5** | SLC12A8 | solute carrier family 12, member 8 | CCC9 | 3q21.2 | - | Ion transport, Potassium ion transport, Transmembrane transport |
| **6** | KLKB1 | kallikrein B, plasma (Fletcher factor) 1 | PPK; KLK3 | 4q35 | Complement and coagulation cascades | Factor XII activation, Proteolysis  Plasminogen activation |
| **7** | ANKHD1 | ankyrin repeat and KH domain containing 1 | MASK; VBARP; PP2500 | 5q31.3 | - | RNA binding, Protein binding, Cytoplasm |
| **8** | SYTL3 | synaptotagmin-like 3 | SLP3 | 6q25.3 | - | Protein binding, Intracellular protein transport, Rab GTPase binding |
| **9** | MYOM2 | myomesin 2 | TTNAP | 8p23.3 |  | Muscle contraction, Structural constituent of muscle, Myosin filament |
| **10** | FAM154A | family with sequence similarity 154, member A | C9orf138 | 9p22.1 | - | - |
| **11** | KIAA1217 | KIAA1217 | SKT; RP11-324E23.1 | 10p12.31 | - | - |
| **12** | FIBIN | fin bud initiation factor homolog (zebrafish) | MGC24932 | 11p14.2 | - | - |
| **13** | LDLRAD3 | low density lipoprotein receptor class A domain containing 3 | LRAD3 | 11p13 | - | Receptor activity |
| **14** | NRXN2 | neurexin 2 | FLJ40892; KIAA0921 | 11q13 | Cell adhesion molecules (CAMs) | Cell adhesion, Neurotransmitter secretion, Synaptic transmission |
| **15** | PROSER1 | proline and serine rich 1 | C13orf23 | 13q13.3 | - | - |
| **16** | KARS | lysyl-tRNA synthetase | KRS; KARS1; KARS2; CMTRIB; DFNB89 | 16q23.1 | Aminoacyl-tRNA biosynthesis, Lysine biosynthesis | Lysine-tRNA ligase activity, Lysyl-tRNA aminoacylation, Metal ion binding |
| **17** | C17orf82 | chromosome 17 open reading frame 82 | x | 17q23.2 | - | - |
| **18** | LAMA3 | laminin, alpha 3 | E170; LOCS; BM600; LAMNA; lama3a | 18q11.2 | ECM-receptor interaction, Small cell lung cancer, Focal adhesion | Laminin-1 complex, Regulation of cell migration, Regulation of cell adhesion |
| **19** | MPND | MPN domain containing | FLJ14981 | 19p13.3 | - | Peptidase activity |
