# Supplementary material for: Biological network inferences for a protection mechanism against familial Creutzfeldt-Jakob disease with E200K pathogenic mutation
Source: BMC Med Genomics. 2014 Aug 22;7:52. doi: 10.1186/1755-8794-7-52 (PMC4151374; doi:10.1186/1755-8794-7-52)
Supplement: Additional file 3: Table S3 — The information of 19 validated sites. Five sites that were false-positive were not recorded in this table. "-" means that there is no data for dbSNP 138. East Asians, Americans, Europeans, Africans were denoted as ASN, AMR, EUR, and AFR respectively. Chromosome, reference alleles and observed alleles in this study were denoted as Chr., Ref., and Obs. respectively. [file 1755-8794-7-52-S3.doc]

Additional file 3: Table S3. The information of 19 validated sites. Five sites which were false-positive were not recorded in this table. “-“ means that there is no data for dbSNP 138. East Asians, Americans, Europeans, Africans were denoted as ASN, AMR, EUR, and AFR respectively. Chromosome, reference alleles and observed alleles in this study were denoted as Chr., Ref., and Obs. respectively

|  | **Chr.** | **Position** | **Gene** | **Ref. /Obs.** | **rs number** | **Created in build** | **Allele frequency in observed population** |
| --- | --- | --- | --- | --- | --- | --- | --- |
| **Validated Sites** | 1 | 27,268,000 | NUDC | G/A | rs140696208 | dbSNP 134 | ASN (G: 99%, A: 1%) |
|  | 1 | 42,049,603 | HIVEP3 | C/T | rs200939032 | dbSNP 137 | ASN (C: 99%, T: 1%) |
|  | 2 | 64,199,317 | VPS54 | G/A | rs140024519 | dbSNP 134 | ASN (G: 96%, A: 4%) |
|  | 2 | 233,346,498 | ECEL1 | C/T | rs142492002 | dbSNP 134 | AMR (C: 99%, T: 1%); ASN (C: 95%, T: 5%) |
|  | 3 | 124,896,625 | SLC12A8 | A/G | rs201533072 | dbSNP 137 | AMR (A: 98%, G: 2%), ASN (A: 99.8%, G: 0.2%) |
|  | 4 | 187,153,290 | KLKB1 | C/T | rs148277206 | dbSNP 134 | ASN (C: 98%, G: 2%) |
|  | 5 | 139,884,478 | ANKHD1-EIF4EBP3 | G/C | rs181178626 | dbSNP 135 | ASN (G: 99%, C: 1%) |
|  | 6 | 159,185,617 | SYTL3 | T/C | rs188114564 | dbSNP 135 | ASN (T: 99.7%, C: 0.3%) |
|  | 8 | 2,088,717 | MYOM2 | G/T | rs143646514 | dbSNP 134 | AMR (G: 99%, T: 1%), ASN (G: 98%, T: 2%),  EUR (G: 99.6%, T: 0.4%), |
|  | 9 | 18,950,859 | FAM154A | C/T | rs201537958 | dbSNP 137 | ASN (C: 99.7%, T: 0.3%) |
|  | 10 | 24,831,649 | KIAA1217 | C/T | rs202157298 | dbSNP 137 | - |
|  | 11 | 27,016,411 | FIBIN | A/G | rs149580940 | dbSNP 134 | ASN (A: 96.5%, G: 0.35%) |
|  | 11 | 36,250,774 | LDLRAD3 | G/T | rs148471178 | dbSNP 134 | AMR (G: 99%, T: 1%), ASN (G: 99%, T: 1%),  AFR (G: 99.8%, T: 0.2%) |
|  | 11 | 64,453,195 | NRXN2 | C/A | - | - | - |
|  | 13 | 39,588,100 | PROSER1 | G/A | COSM1188702 | COSMIC project | - |
|  | 16 | 75,669,878 | KARS | A/G | rs150529876 | dbSNP 134 | ASN (A: 99%. G: 1%) |
|  | 17 | 59,489,425 | C17orf82 | C/G | rs149881215 | dbSNP 134 | ASN (C: 99%. G: 1%) |
|  | 18 | 21,485,578 | LAMA3 | G/C | - | - | - |
|  | 19 | 4,359,190 | MPND | C/T | rs200972486 | dbSNP 137 | ASN (C: 99.7%. T: 0.3%) |
